# Circulating microRNAs as Potential Diagnostic and Prognostic Biomarkers in Hepatocellular Carcinoma

**DOI:** 10.1038/s41598-019-46872-8

**Published:** 2019-07-18

**Authors:** Yu Jin, Ye Shen Wong, Brian K. P. Goh, Chung Yip Chan, Peng Chung Cheow, Pierce K. H. Chow, Tony K. H. Lim, George B. B. Goh, Thinesh Lee Krishnamoorthy, Rajneesh Kumar, Tze Pin Ng, Samuel S. Chong, Hwee Huang Tan, Alexander Y. F. Chung, London Lucien P. J. Ooi, Jason P. E. Chang, Chee Kiat Tan, Caroline G. L. Lee

**Affiliations:** 1 Division of Cellular & Molecular Research, National Cancer Centre Singapore, Singapore, Singapore; 2 Department of Hepato-pancreato-biliary & Transplant Surgery, Singapore General Hospital, Singapore, Singapore; 3 Division of Surgical Oncology, National Cancer Centre Singapore, Singapore, Singapore; 4 Department of Pathology, Singapore General Hospital, Singapore, Singapore; 5 Department of Gastroenterology & Hepatology, Singapore General Hospital, Singapore, Singapore; 6 Department of Psychological Medicine, Yong Loo Lin School of Medicine, National University of Singapore, Singapore, Singapore; 7 Department of Paediatrics, Yong Loo Lin School of Medicine, National University of Singapore, Singapore, Singapore; 8 Department of Laboratory Medicine, National University Hospital, Singapore, Singapore; 9 Blood Services Group, Health Sciences Authority, Singapore, Singapore; 10 Duke-NUS Graduate Medical School, Singapore, Singapore; 11 Department of Biochemistry, National University of Singapore, Singapore, Singapore

**Keywords:** Cancer genomics, Biomarkers

## Abstract

Hepatocellular carcinoma (HCC) is the fifth most common cancer with high mortality, due to late diagnosis and limited treatment options. Blood miRNAs, which circulate in a highly stable, cell-free form, show promise as novel potential biomarkers for early detection of HCC. Whole miRNome profiling was performed to identify deregulated miRNAs between HCC and normal healthy (NH) volunteers. These deregulated miRNAs were validated in an independent cohort of HCC, NH and chronic Hepatitis B (CHB) volunteers and finally in a 3^rd^ cohort comprising NH, CHB, cirrhotic and HCC volunteers to evaluate miRNA changes during disease progression. The associations between circulating miRNAs and liver-damage markers, clinicopathological characteristics and survival outcomes were analysed to identify prognostic markers. Twelve miRNAs are differentially expressed between HCC and NH individuals in all three cohorts. Five upregulated miRNAs (miR-122-5p, miR-125b-5p, miR-885-5p, miR-100-5p and miR-148a-3p) in CHB, cirrhosis and HCC patients are potential biomarkers for CHB infection, while miR-34a-5p can be a biomarker for cirrhosis. Notably, four miRNAs (miR-1972, miR-193a-5p, miR-214-3p and miR-365a-3p) can distinguish HCC from other non-HCC individuals. Six miRNAs are potential prognostic markers for overall survival.

## Introduction

Liver cancer, particularly hepatocellular carcinoma (HCC) is the 5^th^ most common cancer in the world with a poor 5-year survival, of only ~5%, making it the 3^rd^ most deadly cancer^1^. The poor prognosis for HCC patients is mainly due to the late diagnosis of the disease. Major risk factors include Hepatitis B virus (HBV), Hepatitis C virus (HCV), aflatoxin exposure, alcoholic disease and nonalcoholic fatty liver disease (NAFLD)^2^. A great majority (70–90%) of HCC patients suffers from liver cirrhosis^3^.

Diagnosis of HCC at an early stage can facilitate the effective treatment and improve the survival. Conventionally, HCC is diagnosed using a combination of hepatic ultra-sound imaging and serum α-fetoprotein (AFP)^4^. However, as a serum biomarker, AFP lacks specificity^5^. Great efforts have been made to develop biomarkers that could provide high diagnostic accuracy and benefit effective treatment and surveillance.

The genetic basis of HCC has always garnered researchers’ interests. Aberrant miRNA expression has been identified in a variety of human cancers including HCC^6^. Since an early study by Murakami *et al*. in 2006^7^, which identified 3 up- and 5 down-regulated miRNAs, there have been a number of studies profiling whole miRNome-wide miRNA expression in HCC, and similar number of up- and down-regulated miRNAs were reported in HCC tissues^8^. Commonly de-regulated miRNAs include down-regulated let-7 family^8^ and miR-122^9–11^ as well as up-regulated MiR-221, miR-222 and miR-224^10,12,13^.

The signatures of aberrant miRNA expression in HCC tissues lead to the exploration of these miRNAs as diagnostic and prognostic markers for HCC. However, the use of tissue miRNA expression for diagnosis is not as practical as less invasive markers e.g. serum AFP. Instead, circulating miRNAs in body fluids e.g. plasma, urine are considered as the potential candidates for biomarkers. miRNAs have been detected in the body fluids including plasma at relatively stable levels and hence has potential to distinguish cancer patients from normal individuals^14^. Although circulating miRNAs as non-invasive biomarkers have only emerged recently, a number of studies have already demonstrated their potential as biomarker in a variety of cancers including lung cancer, prostate cancer and gastric cancer^15^.

Independent research groups had investigated circulating miRNA de-regulation in HCC. However, most of these studies focused on a single or several candidate circulating miRNAs, based on the knowledge of their implications in HCC or other cancers. A few miRNAs have been extensively studied, including miR-21, which are also de-regulated in other cancers^16^ and show consistent up-regulation in HCC^17–19^, suggesting that they may serve as general cancer biomarkers. Circulating miR-122 has been associated with both HCC and liver pathologies although reports were inconsistent. Two genome-wide profiling studies identified that miR-122 was under-expressed in HCC serum or plasma samples compared to normal control group^17,20^. However, Xu *et al*. and Qi *et al*. observed up-regulation of circulating miR-122 in HCC compared to normal group^18,21^. By integrating the miRNA expression in plasma samples of CHB, it was observed that miR-122 is significantly elevated during chronic HBV infection, hence miR-122 is a good candidate biomarker for early liver pathology, but not specifically for HCC^21^. MiR-223 expression was also examined in HCC and liver diseases in several studies, but the trends of de-regulation remained inconsistent. Down-regulation of miR-223 was observed in HCC, CHB and cirrhotic patients^17,22^. However, contradictory findings by Xu *et al*. and Wang *et al*. indicated that the same miRNA was up-regulated in HCC and/or CHB patients^18,23^.

Thus far, most studies on circulating miRNAs do not examine changes in miRNA expression during disease progression from healthy to CHB to cirrhotic to HCC and are focused mainly on selected miRNAs. Here, we report the comprehensive genome-wide profiling of circulating miRNA to identify potential biomarkers that may distinguish different disease states of CHB, cirrhosis and HCC as well as prognostic biomarkers for survival and other clinical characteristics.

## Results

### Twelve miRNAs consistently differentially de-regulated in HCC

miRNA expression profiles between HCC and NH were examined in all three cohorts. Whole miRNome profiling of 19 HCC and 10 NH controls in the discovery phase identified 34 significantly up- and 22 significantly down-regulated miRNAs between the two groups (fold change > 2 and corrected P-value < 0.05 by Student’s t-test). MiR-100-5p, was identified as an up-regulated miRNA as it was expressed in 70% of HCC patients but undetectable in NH volunteers.

**Figure 1:**
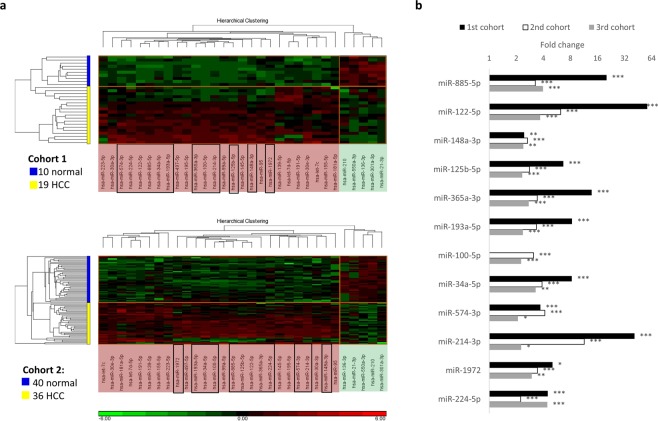
Significantly differentially expressed miRNAs between HCC and normal healthy samples. (**a**) Expression heatmaps of the 31 plasma miRNAs that are significantly de-regulated in HCC (fold change > 2, corrected p-value < 0.05) compared to NH individuals in the first (top panel) and the second cohorts (bottom panel). MiRNAs shaded in red at the horizontal axes were up-regulated in HCC while those shaded in green were down-regulated. Black-line boxed miRNAs were successfully validated in the third cohort (29 NH, 64 HCC). (**b)** Twelve plasma miRNAs that showed consistent and significant difference in all the three cohorts. *Corrected p-value < 0.05, **corrected p-value < 0.01, ***corrected p-value < 0.001.

Of the 56 de-regulated miRNAs, 26 up and five down-regulated miRNAs were successfully validated in the second cohort comprising 36 HCC and 40 NH samples (Fig. 1a). Further analyses of these de-regulated miRNAs in a third cohort comprising 64 HCC patients and 29 NH controls revealed only 12 up-regulated miRNAs were consistently significantly differentially expressed in all the three cohorts (Fig. 1b).

**Figure 2:**
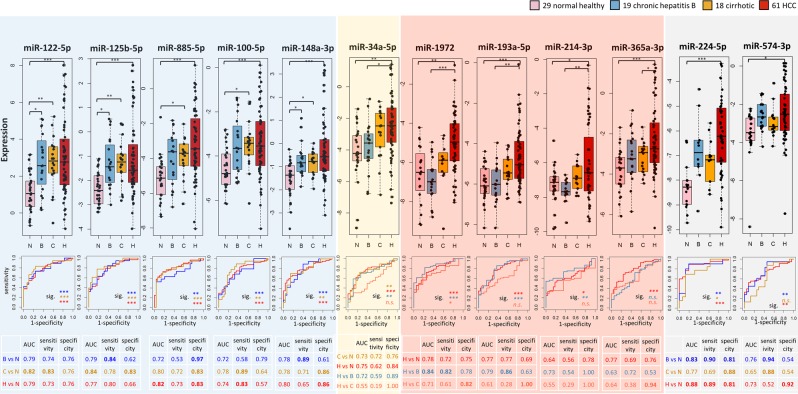
Expression patterns and ROC curves of the 12 consistently up-regulated miRNAs between HCC and NH. Top panel: boxplots of the expression levels of the 12 circulating miRNAs in normal healthy (N), CHB (B), cirrhotic (C) and HCC (H) samples. Pairwise comparison of expression levels with statistically significant difference were indicated in the box plot. ***Corrected p-value < 0.001, **corrected p-value < 0.01, *corrected p-value < 0.05. Bottom panel: ROC plots of the twelve miRNAs for distinguishing different disease groups and NH samples. Statistical significance of the ROC analysis were indicated under the curves. AUC, sensitivity and specificity were summarized in the tables below ROC plots. Sig. significance level. ***p-value < 0.001, **p-value < 0.01, *p-value < 0.05. n.s. not significant.

We then proceeded to evaluate the change of expression of these 12 consistently up-regulated miRNAs across different disease states from healthy to chronic Hepatitis B (CHB) infected to cirrhotic and finally to HCC using plasma samples from the 3^rd^ cohort of patients to determine their expression changes during disease progression. Five of these 12 miRNAs (miR-122-5p, miR-125b-5p, miR-100-5p, miR-885-5p and miR-148a-3p) may be good biomarkers for liver pathologies as they were also significantly up-regulated in CHB and cirrhotic samples compared to NH, but not amongst samples with liver pathologies i.e. CHB, cirrhosis and HCC (Fig. 2, shaded blue). From the receiver operating characteristic (ROC) analysis, miR-122-5p and miR-125b-5p showed the best diagnostic performance for detection of liver pathologies (Fig. 2, bottom of leftmost 2 blue panels) as they were able to significantly distinguish samples with liver pathologies from NH group (p-values < 0.001 by Mann-Whitney test). MiR-885-5p and miR-100-5p were good markers for cirrhosis however were less sensitive (sensitivity < 0.6) for distinguishing the CHB from NH group (Fig. 2, bottom of 3^rd^ and 4^th^ blue panels). Circulating miR-34a-5p is a potential cirrhotic biomarker (Fig. 2, shaded yellow) as it is significantly elevated in cirrhotic/HCC compared to NH/CHB patients. ROC analysis showed fair performance for distinguishing cirrhotic from NH groups (area under curve AUC = 0.73), but poor performance for diagnosis of HCC from cirrhotic samples (AUC = 0.55). Therefore, miR-34a-5p was suitable for detection of cirrhosis preceding HCC.

Notably, four miRNAs (miR-1972, miR-193a-5p miR-214-3p and miR-365a-3p) were identified to be specifically and consistently up-regulated only in HCC patients but not amongst the three (cirrhotic, CHB and NH) groups of non-HCC (Fig. 2, shaded in red). Non-HCC (NH, CHB and cirrhotic) patients showed similar expression levels of these plasma miRNAs. ROC analyses revealed that miR-1972 displayed the best diagnostic performance for distinguishing HCC from the non-HCC groups, while miR-193a-5p, miR-214-3p and miR-365a-3p showed good AUC for differentiating HCC from CHB or NH groups, but does not clearly distinguish HCC from cirrhotic patients (AUC < 0.7, p-values > 0.05), perhaps due to the low sample size for cirrhotic (n = 18) patients. Further analyses in a larger cohort of (especially cirrhotic) patients will help clarify their usefulness as specific HCC biomarkers.

Lastly, we observed that though circulating miR-574-3p and miR-224-5p showed higher expression in CHB and HCC compared to NH group, their expression levels were lower though insignificant in cirrhotic compared to CHB samples (Fig. 2, shaded grey). However, as their trend of expression was not consistent from NH to CHB to cirrhosis to HCC, these 2 miRNAs may not be appropriate biomarkers for liver pathologies or HCC.

In summary, 12 miRNAs were consistently up-regulated in the plasma of HCC compared to NH individuals. Five (miR-122-5p, miR-125b-5p, miR-885-5p, miR-100-5p and miR-148a-3p) of these may serve as potential biomarkers for liver disease as significantly up-regulation in these miRNAs was observed between NH and patients with liver disease (CHB, cirrhotic and HCC) but not amongst patients with liver disease (CHB, cirrhotic and HCC) (Fig. 2, shaded blue). MiR-34a-5p may serve as a potential cirrhotic biomarker as it is elevated in cirrhotic patients with no further increase in HCC patients (Fig. 2, shaded yellow). Notably, miR-1972, miR-193a-5p, miR-214-3p and miR-365a-3p are potentially specific HCC biomarkers as their expressions are similar in non-HCC (NH, CHB and cirrhotic) but significantly elevated only in HCC patients.

### Clinical characteristics associated with overall survival in HCC patients

**Figure 3:**
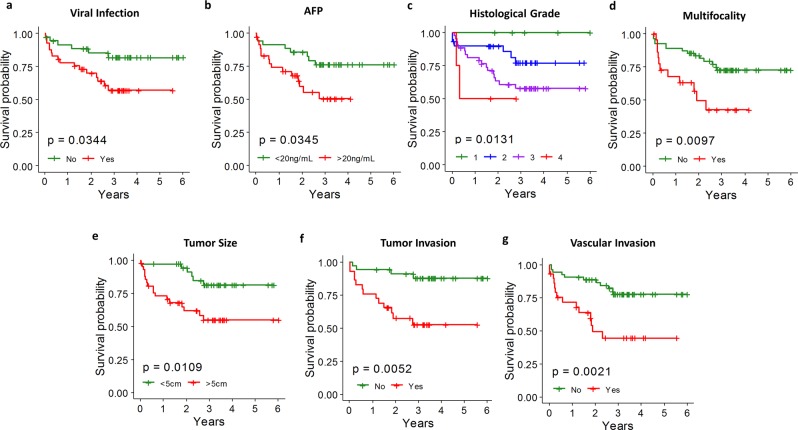
Clinical characteristics associated with overall survival. (**a**) Viral infection was associated with worse overall survival outcome. (**b**) High AFP level (>20 ng/mL) was associated with worse overall survival. (**c**) Increasing Edmondson’s histological grade correlated with decreasing overall survival time. (**d**) Multifocality was associated with worse overall survival outcome. (**e**) Large tumor size (>5 cm) was associated with short overall survival time. **(f)** Tumor invasion was associated with worse overall survival outcome. (**g**) Vascular invasion was associated with worse overall survival outcome.

The clinical features of the 116 HCC patients included in this study were summarized in Table 1. We first investigated their associations with overall survival and identified seven features significantly correlating with overall survival outcome by Cox proportional hazards regression model (p-value < 0.05). Presence of viral infection and increasing serum AFP level were associated with worse survival outcomes (Fig. 3a,b, red lines). None of the non-tumor characteristics i.e. normal liver cirrhosis, steatosis and dysplasia could contribute to survival outcomes (p-value > 0.05, Table S2). Tumor characteristics including increasing Edmondson’s histological grade, presence of multiple tumor nodules, increasing tumor size, tumor invasion and vascular invasion were all associated with shorter overall survival time (Fig. 3c–g).

**Table 1:** Summary of clinical characteristics of HCC patients.

Clinical characteristics	Phenotypes	# HCC patients	%
Gender	Male	100	86%
	Female	16	14%
Age		63.3 ± 11.1	
Race	Chinese	104	90%
	Malay	4	3%
	Indian	1	1%
	Others	6	5%
Viral infection	HBV	55	56%
	HCV	6	6%
	None	38	38%
Normal liver cirrhosis	Yes	52	54%
	No	44	46%
Steatosis	Yes	35	70%
	No	15	30%
Dysplasia	Yes	7	15%
	No	41	85%
Histological Grade	1	6	7%
	2	35	38%
	3	47	51%
	4	4	4%
Multifocality	Multi-nodal	27	29%
	Single nodal	65	71%
Tumor Size (cm)		6.8 ± 4.3	
Tumor Encapsulation	Yes	54	58%
	No	39	42%
Degree of Encapsulation	Complete	18	36%
	Incomplete	32	64%
Hepatic Capsule	Tumor Present	15	16%
	Tumor Free	79	84%
Local extension	Tumor invades	7	8%
	Confined to liver	86	92%
Tumor Necrosis	Yes	61	66%
	No	32	34%
Perineural Invasion	Yes	3	3%
	No	87	97%
Tumor Invasion	Yes	30	43%
	No	39	57%
Vascular Invasion	Yes	34	36%
	No	60	64%

### Twenty miRNAs are associated with liver damage markers, clinical characteristics and/or overall survival

The value of plasma miRNAs as prognostic biomarker was explored, by associating plasma miRNAs with various clinical characteristics. Twenty miRNAs showed statistically significant associations (corrected p-value < 0.05) with various liver damage/HCC markers like aspartate aminotransferase (AST) and AFP as well as clinically relevant characteristics (Table 2). Amongst the tumor-associated characteristics, only tumor invasion was significantly associated with increasing levels of three miRNAs, let-7a-5p, miR-320d, and miR-423-5p in the plasma samples of HCC patients. Two non-tumor characteristics, steatosis and cirrhosis, were found to be significantly associated with circulating miRNA expression in these HCC patients. HCC patients with cirrhosis in their non-tumor liver were found to express significant higher levels plasma miR-451a and miR-222-3p, and lower levels of miR-483-5p, miR-574-3p, miR-193a-5p and miR-152 compared to patients without normal liver cirrhosis. Notably, both miR-574-3p and miR-193a-5p were consistently up-regulated in HCC and compared to NH volunteers (Fig. 1b) while miR-193a-5p was also found to be a potential HCC biomarker (Fig. 2, shaded red). Similarly HCC patients with steatosis in the non-tumorous liver expressed significantly lower levels of plasma miR-214-3p and miR-95 (Table 2). miR-214-3p has potential to be a biomarker that can distinguish HCC from non-HCC individuals (Fig. 2, shaded red).

**Table 2:** Circulating miRNAs associated with clinical characteristics.

	MiRNA	Non-Tumor Liver				General						Tumor							
		Steatosis		Cirrhosis		AST		AFP		Viral Infection		Tumor Size		Tumor Invasion		Multifocality		Survival	
		Fold change (Present/Absent)	p-value*	Fold change (Present/Absent)	p-value^*^	Rank correlation coefficient	p-value^*^	Rank correlation coefficient	p-value^*^	Fold change (Present/Absent)	p-value	Rank correlation coefficient	p-value	Fold change (Present/Absent)	p-value^*^	Fold change (Multi-/Single-nodal)	p-value	Hazard Ratio	p-value^*^
1	miR-214-3p	**−3**.**96**	**1**.**78** × **10**^**−2**^																
2	miR-95	**−3**.**36**	**3**.**52** × **10**^**−5**^																
3	miR-451a			**3**.**42**	**1**.**31** × **10**^**−2**^			**−0**.**46**	**9**.**05** × **10**^**−8**^										
4	miR-222-3p			**1**.**64**	**1**.**33** × **10**^**−2**^														
5	miR-483-5p			**−2**.**29**	**3**.**75** × **10**^**−3**^			**0**.**46**	**2**.**95** × **10**^**−6**^										
6	miR-574-3p			**−2**.**16**	**1**.**27** × **10**^**−4**^														
7	miR-193a-5p			**−2**.**06**	**1**.**31** × **10**^**−2**^														
8	miR-152			**−1**.**56**	**3**.**75** × **10**^**−3**^														
9	miR-423-3p							**0**.**31**	**5**.**12** × **10**^**−4**^										
10	miR-221-3p							**0**.**23**	**1**.**23** × **10**^**−2**^										
11	let-7b-5p							**−0**.**34**	**8**.**84** × **10**^**−5**^										
12	miR-301a-3p							**−0**.**23**	**1**.**30** × **10**^**−2**^										
13	miR-34a-5p					**0**.**34**	**2**.**75** × **10**^**−2**^												
14	miR-29a-3p					**0**.**31**	**2**.**75** × **10**^**−2**^												
15	miR-22-5p					**0**.**31**	**2**.**75** × **10**^**−2**^												
16	let-7a-5p													**2**.**00**	**4**.**11** × **10**^**−2**^				
17	miR-320d													**1**.**59**	**3**.**58** × **10**^**−2**^				
18	miR-423-5p													**1**.**56**	**3**.**58** × **10**^**−2**^				
19	miR-410							**0**.**32**	**2**.**27** × **10**^**−3**^	1.71	2.11 × 10^−2^							**1**.**79**	**9**.**42** × **10**^**−3**^
20	miR-382-5p							**0**.**24**	**1**.**74** × **10**^**−2**^			0.21	4.53 × 10^−2^					**1**.**64**	**9**.**42** × **10**^**−3**^
21	miR-139-5p													1.62	3.34 × 10^−2^	1.58	3.15 × 10^−2^	**1**.**77**	**9**.**42** × **10**^**−3**^
22	miR-128											0.20	4.63 × 10^−2^	1.47	4.84 × 10^−2^			**2**.**35**	**9**.**65** × **10**^**−3**^
23	miR-101-3p											−0.24	1.72 × 10^−2^	−2.19	3.50 × 10^−2^			**0**.**75**	**9**.**42** × **10**^**−3**^
24	miR-424-5p									−1.66	1.24 × 10^−2^	−0.22	2.85 × 10^−2^	−1.87	7.12 × 10^−3^			**0**.**69**	**9**.**65** × **10**^**−3**^

^*^Bold indicates corrected p-values that are < 0.05 i.e. these miRNAs show statistically significant association after multiple test corrections, while non-bold shows p-values before multiple test correction.

Eleven miRNAs were significantly associated with the biochemical liver damage/HCC markers, AFP or AST, respectively (Table 2). miR-34a-5p was also positively correlated with the liver damage marker AST (Table 2) and found to be a potential biomarker for liver cirrhosis (Fig. 2). Amongst the eight miRNAs that significantly correlated with AFP levels, miR-410 and miR-382-5p were also positively correlated overall survival in addition to being associated with AFP (Table 2).

### Six circulating miRNAs as potential prognostic markers for overall survival

**Figure 4:**
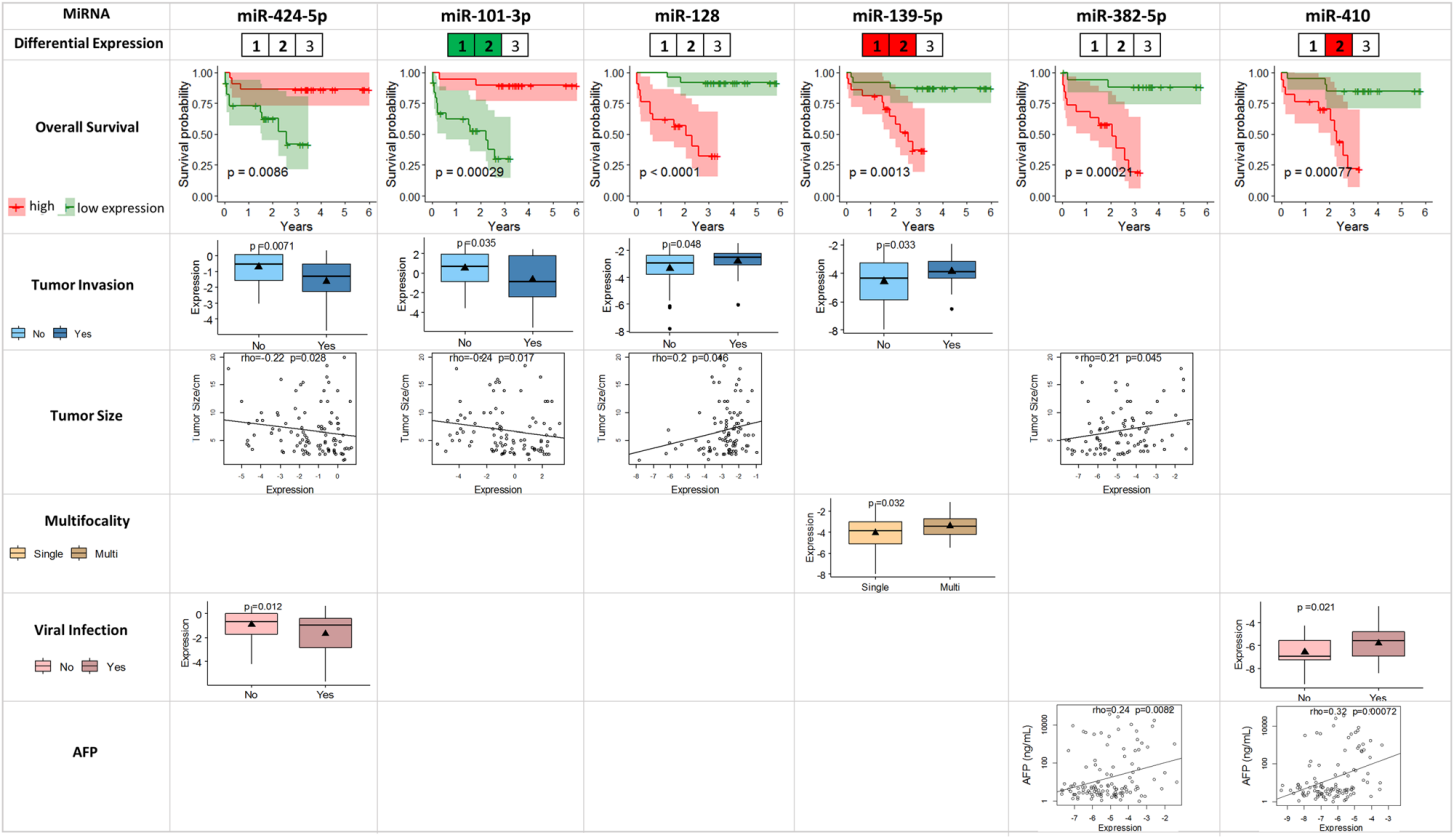
Six plasma miRNAs associated with overall survival outcome and clinical characteristics including tumor invasion, tumor size, multifocality, viral infection and AFP level. Differential expression: the three cells labelled as 1, 2, 3 corresponds to the miRNA expression difference between HCC and NH in the first (10 NH, 19 HCC), second (40 NH, 36 HCC) and the third (29 NH, 61 HCC) cohorts, respectively. MiRNAs significantly up-regulated in a specific cohort was highlighted in red and miR-101-3p significantly down-regulated in the first and the second cohorts were highlighted in green. Overall survival: based on the first and the third quartiles of each miRNA’s expression, the patients were classified into “high-expression” and “low-expression” groups, and the Kaplan-Meier curves for these two groups were plotted for the each of the miRNAs significantly associated with overall survival. The shaded areas represent the 95% confidence interval. Tumor invasion: boxplots for the plasma miRNAs differentially expressed between patients with and without tumor invasion (p-value < 0.05 by Student’s t-test) were plotted. (p-value < 0.05 by Student’s t-test) were plotted. Tumor size: dot plots for the miRNAs showing significant correlation with tumor size (p-value < 0.05 by Spearman’s rank correlation test) were plotted with the trend lines. Multifocality: boxplots for the plasma miRNAs differentially expressed between patients with single and multiple nodules (p-value < 0.05 by Student’s t-test) were plotted. Viral infection: boxplots for the plasma miRNAs differentially expressed between patients with and without virus infection (p-value < 0.05 by Student’s t-test) were plotted. AFP: dot plots for the miRNAs showing significant correlation with serum AFP level (p-value < 0.05 by Spearman’s rank correlation test) were plotted with the trend lines.

Survival outcomes are of great interests to patients, doctors as well as policy makers as they represent measures to estimate the prognosis and course of the disease of the cancer patients. Here, we evaluated the association between plasma miRNA expression levels and overall survival outcome in 116 HCC patients from all three cohorts of HCC patients, using univariate Cox proportional hazards regression model. Six miRNAs showed significant association with overall survival outcomes (corrected p-value < 0.05), including miR-410 and miR-382-5p which were also positively correlated with serum AFP level as well (corrected p-value < 0.05) (Table 2). Among the six survival-associated miRNAs, higher expression of four miRNAs (miR-128, miR-139-5p, miR-382-5p and miR-410) corresponded to worse survival outcome while the other two miRNAs (miR-424-5p and miR-101-3p) showed lower expression level in the worse prognosis groups (Fig. 4). None of these six miRNAs was consistently de-regulated in HCC patients compared with normal health control volunteers in all the three cohorts.

Not only is lower expression of miR-424-5p and miR-101-3p significantly correlated with worse survival outcomes (corrected p-value < 0.05), they are also similarly correlated with tumor invasion (p < 0.05, Student’s t-test) and tumor size (p < 0.05, Spearman’s rank correlation test) (Fig. 3, Table 2). Thus, miR-424-5p and miR-101-3p may play a role in overall survival through modulating tumor size and tumor invasion.

On the other hand, high expression of miR-128 is also correlated with larger tumors and tumor invasion; while higher miR-139-5p expression is found in HCC patients with multiple tumor nodules and tumor invasion, both leading to significantly poorer overall survival (Fig. 3, Table 2). In addition to being associated with significantly poorer overall survival, high miR-382-5p is also associated with larger tumors as well as significantly higher levels of the HCC biomarker, serum AFP while higher miR-410 is also associated with HCC patients with viral infection and significantly higher serum AFP (Fig. 3, Table 2).

## Discussion

HCC is a good yet challenging disease for the development of biomarkers as it largely occurs in a background of hepatitis and cirrhosis^24^. Although different potential biomarkers have been identified for HCC, they mainly detect late but not early HCC.

An ideal HCC biomarker should be sensitive, specific, reproducible, affordable and non-invasive, being able to be evaluated in readily accessible tissues like body fluids, including serum, plasma, urine or saliva. miRNAs are attractive as potential less/non-invasive biomarkers since they circulate in a highly stable, cell-free form in the blood^14^ and even saliva^25,26^.

Here, plasma miRNAs was profiled in 4 different groups of patients/volunteers (NH, CHB, cirrhotic and HCC) to identify miRNA biomarkers whose expression progresses through different stages from normal healthy individuals to HCC development, which have the potential to identify biomarkers of HCC at an early stage. Additionally, miRNAs were correlated with various clinical characteristics in HCC patients to identify miRNAs that can serve as potential prognostic biomarkers.

By investigating three independent cohorts comprising a total 116 HCC patients and 79 NH individuals, we identified 12 plasma miRNAs that are consistently up-regulated in HCC compared to normal controls. Five of these (miR-122-5p, miR-100-5p, miR-125b-5p, miR-885-5p and miR-148a-3p) have potential to serve as biomarkers for the early detection of liver pathologies since their expression are significantly increased between CHB and NH with no further increase in cirrhotic and HCC patients. Of these, miR-122-5p and miR-125b-5p exhibited the best diagnostic performance (Fig. 2, bottom panel, shaded blue).

Another 4 of these 12 miRNAs (miR-1972, miR-193a-5p, miR-214-3p and miR-365a-3p) that are consistently up-regulated in HCC patients compared to NH were found to have potential to be good HCC biomarkers as these miRNAs are significantly upregulated in HCC patients but no significant difference was observed amongst the non-HCC patients (Fig. 2, shaded red). Of these, the novel miR-1972 has the best diagnostic performance (Fig. 2, bottom panel, shaded in red). While miR-214-3p and miR-365a-3p were found to be up-regulated in the plasma of HCC patients (Fig. 2), both these miRNAs were reported to be expressed in lower levels in tumor versus the adjacent non-tumorous tissues of HCC patients^27,28^.

Consistent with our observation, circulating levels of miR-122-5p^21^, miR-125b-5p^22^, miR-100-5p^23^, miR-885-5p^29^ were also previously reported to be higher in HCC patients compared to normal healthy group. Similarly, circulating levels of miR-122-5p^30^, miR-125b-5p^22^ and miR-885-5p^29,30^ were also previously reported to be up-regulated in various liver pathologies including CHB and cirrhosis. Circulating miR-148a-3p was elevated in patients undergoing liver transplantation^31^.

Circulating miR-34a-5p has potential to be a cirrhotic biomarker (Fig. 2, shaded yellow) and is significantly associated with the expression of AST, a liver damage marker (Table 2). These results are consistent with previous findings of up-regulation of serum miR-34a-5p in patients with cirrhosis and positive correlation between serum miR-34a-5p and AST level^30^.

Consistent with the circulating miRNA trend, miR-125b-5p, which targets the Stard13 protein to mediate miR-125b-Stard13-RhoA-α-SMA signaling cascade involved in hepatic stellate cell activation during fibrosis, was also found to be up-regulated in fibrotic liver tissues^32^. Similarly, miR-148a-3p, which targets and inhibits PTEN leading to the activation of β-catenin signalling, was reported to be up-regulated in HBx positive liver samples from patients infected with HBV^33^.

However, curiously, several of the miRNAs, whose expression was increased in the plasma/serum of HCC patients were reported to be expressed at lower levels in HCC tumor compared to non-tumorous tissues. Although miR-122-5p, which is involved in lipid metabolism^34^, hepatocyte differentiation^35^, mitochondrial metabolism^10^ as well as HBV gene expression^36^, is the most abundantly expressed miRNA in the liver, its expression was reported to be down-regulated in HCC tumor^9–11^. Similarly, miR-885-5p, which targets CTNNB1 and modulates Wnt/β-catenin signaling pathway, was down-regulated in highly malignant HCC tumor tissues^37^. CTNNB1 was also reported to be indirectly targeted by miR-214-3p, which was found to be suppressed in HCC tumor tissues^38^. Likewise, miR-100-5p, which modulates apoptosis and cell growth by targeting plk1, was reported to be down-regulated in HCC tumor and its low expression was associated with worse prognosis^39^. miR-365a-3p, which targets Bcl-2 to regulate cell apoptosis, was also reported to be down-regulated in HCC tissues^40^.

Contradictory observations have been reported about miR-193a-5p in the tissues of HCC patients. Wang *et al*. reported that miR-193a-5p, which targets BMF to modulate cell proliferation, G1/S transition and apoptosis, was more highly expressed in the tumors compared to the non-tumor tissues of HCC patients^41^. However, Li *et al*. reported that miR-193a-5p, targeting SPOCK1, was down-regulated in HCC patients^42^.

In addition to identifying miRNAs that are deregulated during the progression of disease from healthy to chronic hepatitis to cirrhotic to HCC patients, the profile of plasma miRNAs expression was also associated with various clinical characteristics, survival outcomes and/or liver damage/HCC marker expression of the HCC patients (Table 2) to identify potential prognostic biomarkers. Majority of the 24 plasma miRNA associated with HCC patient characteristics were significantly correlated with non-tumor characteristics including cirrhosis, steatosis and liver disease markers. Notably, the potential cirrhotic biomarker, miR-34a-5p (Fig. 2, shaded yellow), was also positively correlated with liver damage marker AST (corrected p < 0.05) (Table 2) as discussed above. Curiously, 3 miRNAs (miR-214-3p, miR-574-3p and miR-193a-5p) which are consistently up-regulated in HCC patients compared to NH (Fig. 1b), of which 2 (miR-214-3p and miR-193a-5p) have potential to be HCC specific biomarkers (Fig. 2), were also found to be inversely associated (corrected p < 0.05) with steatosis (miR-214-3p) and cirrhosis (miR-574-3p and miR-193a-5p) of the non-tumorous part of the liver in HCC patients.

HCC patients with tumor invasion were found to express significantly higher plasma levels of 3 miRNAs (let-7a-5p, miR-320d and miR-423-5p) than those without tumor invasion (Table 2). Consistent with our findings, high levels of let-7a in tumor tissues were reported to be associated with serosal and vein invasion^43^. Similarly, over-expression of miR-423 was reported to promote cell invasion in HCC cell line^44^. Hence, circulating let-7a and miR-423 can serve as potential prognostic biomarker for tumor invasion.

Survival outcomes are key parameters to evaluate the prognosis and course of the disease of the cancer patients. Here, we showed that viral infection, high AFP levels as well as tumor characteristics including Edmondson’s histological grade, multiple nodules, larger tumor size, tumor and vascular invasion were significantly correlated with worse overall survival (Fig. 3).

These observations are consistent with previous studies that identified AFP, virus infection, multiple nodules and vein invasion being associated with overall survival^43,45,46^. It is notable that non-tumor characteristics, including liver cirrhosis did not show significant correlation with overall survival outcome in both the previous study^46^ and this study (Table S2). In addition to the clinical characteristics, six plasma miRNAs (miR-424-5p, miR-101-3p, miR-128, miR-139-5p, miR-382-5p and miR-410) were significantly associated with survival as well as the clinical characteristics that may contribute to overall survival outcomes (Fig. 4). Four out of the six miRNAs were also associated with tumor invasion, including miR-424-5p and miR-101-3p whose down-regulation correlating with presence of tumor invasion and worse survival outcome, as well as miR-128 and miR-139-5p with higher expression in the patients with tumor invasion and worse survival outcome. Down-regulation of circulating miR-424-5p in serum samples of HCC patients was also reported to be associated with poor prognosis in a previous study^47^. miR-424-5p, which was reported to affect growth through inhibiting Akt3 and E2F3^48^, was down-regulated in tumor tissues and its expression levels in HCC tissues correlated with tumor size, multiple nodules, tumor stage and overall survival outcome. Taken together, decreased expression of circulating miR-424-5p is reflective of tumor progression and invasion contributing to shorter survival, and thus would be a promising prognostic marker for HCC patients. Consistent with our findings, the other miRNA, miR-101-3p, whose high expression correlates with favourable prognosis (Fig. 4), was reported to be down-regulated in HCC tumors compared to adjacent non-tumorous tissues and the low expression of tumor miR-101-3p was associated shorter overall survival by inhibiting SOX9^49^. Furthermore, lower expression of plasma miR-101-3p was observed in patients with tumor invasion, and the expression of miR-101-3p was negatively correlated with tumor size (Fig. 4). This is consistent with a previous report which observed lower expression of miR-101 in HCC tumor compared to match normal tissues and the inhibition of proliferation, migration and invasion abilities of HepG2 cells transfected with miR-101^50^. Unlike miR-424-5p and miR-101-3p, higher expression of miR-128 and miR-139-5p, miR-382-5p and miR-410 was found to be significantly associated with worse survival outcome as well as various other tumor characteristics/marker/risk factor including the presence of tumor invasion, larger tumor size, multiple nodules, viral infection or higher AFP level (Fig. 4). Consistent with our observation that high plasma miR-410 leads to worse prognosis, miR-410, was also found to be over-expressed in the tumors of HCC patients and targets FHL1 to enhance cell growth^51^. Curiously miR-128, miR-139-5p and miR-382-5p were reported to be down-regulated in the tumors of HCC patients and low tumor expression of these miRNAs in tumor tissues are associated with poor prognosis^52–55^. It is thus pertinent to further investigate the seeming contradictory observations that higher plasma expressions levels (Fig. 4) but lower tumor levels^52–55^ of these miRNAs are associated with poorer prognosis. Nonetheless, these 6 miRNAs that significantly affect overall survival through modulating various tumor/cancer characteristics represent promising prognostic biomarkers for HCC.

Hence, circulating miRNA expression profiles may not always be consistent with the tumor expression profiles as observed for diagnostic markers including miR-122-5p, miR-100-5p, miR-214-3p, miR-365a-3p, and prognostic markers including miR-128, miR-139-5p and miR-382-5p. A previous study reported that circulating miRNAs can be released from tissues into blood circulation as a result of cell death (either apoptosis or necrosis)^56^. For example, the up-regulation of circulating miR-122-5p in patients with CHB could be due to its release from damaged hepatocytes as a result of inflammation^21^. miRNAs, like miR-148a, can also be released through active exosome secretion^56^ to play roles in cell-cell communication^57^. The molecular mechanism behind the de-regulation of each potential miRNA biomarker thus remains to be fully elucidated.

In conclusion, through the systematic analyses of >700 miRNAs in a total of 262 samples from 3 independent cohorts, we have identified 12 miRNAs that are consistently and significantly expressed at higher levels in HCC patients compared to normal health (NH) volunteers. Five of these miRNAs (miR-122-5p, miR-125b-5p, miR-100-5p, miR-885-5p and miR-148a-3p) are potential chronic HBV infection biomarker, one (miR-34a-5p) is a potential cirrhotic biomarker while 4 (miR-1974, miR-193a-5p, miR-214-3p and miR-365a-3p) can be potential HCC biomarker with miR-1974 showing the best diagnostic performance. Notably, 6 miRNAs (miR-424-5p, miR-101-3p, miR-128, miR-139-5p, miR-382-5p and miR-410) are potential promising prognostic biomarkers as they are significantly associated with overall survival and various pertinent tumor/cancer characteristics. It is thus worthwhile to validate these findings in a larger cohort of patients as well as elucidate the underlying mechanism of the deregulation of circulatory miRNAs.

## Methods

### Sample collection, RNA isolation, miRNA profiling and data processing

Blood from patients with HCC, CHB, cirrhosis and NH were collected with informed consent from the patients and prior approval from the SingHealth Centralized Institutional Review Board (CIRB Refs: 2013/455/B and 2014/823/B). The samples were divided into three cohorts. In the first discovery phase cohort, we compared the circulating miRNA expression between 10 NH and 19 HCC patients. Differentially expressed miRNAs from the first cohort were then validated in the second cohort, which interrogates plasma samples from 40 NH, 36 HCC as well as 30 CHB patients. To evaluate changes in these miRNA expression as the disease progresses from normal to CHB to cirrhotic and finally HCC, these deregulated miRNAs are evaluated in a 3^rd^ cohort comprising 29 NH, 19 CHB, 18 cirrhotic and 61 HCC plasma samples (Table S1). The inclusion criteria and sample collection procedures are described in Supplemental Methods.

Plasma was isolated from whole blood samples after centrifugation as described in the supplemental methods. Total RNA was extracted from 250 μl plasma using the miRCURY™ RNA isolation kit – biofluids (Exiqon, Vedbaek, Denmark) according to the manufacturer’s instructions. A quality control step was conducted prior to whole-miRNome or candidate miRNA profiling (see Supplemental Methods). Samples with low spike-in signals or have a high risk of hemolysis were removed from analyses.

Whole-miRNome profiling using Exiqon microRNA Ready-to-Use PCR Human panels I + II was performed for the samples in the first and the third cohort, and customized Pick&Mix panels were designed for validation purpose and utilized for the samples in the second cohort. For both whole-miRNome, or customized miRNA panel profiling, the isolated total RNA was reverse transcribed into cDNA using the miRCURY LNA™ Universal RT microRNA PCR, Polyadenylation and cDNA synthesis kit (Exiqon). The synthesized cDNA was assayed by qPCR on the microRNA Ready-to-Use panels and ExiLENT SYBR® Green mastermix according to the manufacturer’s protocol. Each miRNA was assayed once in a 10 μl reaction according to the miRCURY LNA™ Universal RT microRNA polymerase chain reaction (PCR) protocol. Amplification was performed in a LightCycler® 480 Real-Time PCR System (Roche), and the quantification cycle (Cq) values were determined by the second derivative method using the Roche LC software. For each of the miRNA assayed, we removed samples whose Cq values are within 5 Cq values of the negative control. Lastly, global mean of common miRNAs was employed to normalize the raw data as NormFinder software found global mean to be more stable than any single miRNA. Global mean normalization was employed to calculate the normalized Cq values, as described in the supplemental methods.

### Statistical analysis

To compare the miRNA expression between biological groups (HCC, CHB, NH, cirrhotic), pairwise Student’s t-test was performed followed by Benjamini-Hochberg multiple test correction. MiRNAs that show fold change more than two and corrected p-value < 0.05 were identified as significantly differentially expressed miRNAs. The heat-maps for the expression profiles of the differentially expressed miRNAs between HCC and NH samples were plotted using Partek Genomic Suite 6.6^58^.

To evaluate the diagnostic performance of the differentially expressed plasma miRNAs for discriminating disease groups from controls, ROC curves were constructed based on a single miRNA expression. AUC, optimal sensitivity and specificity were calculated in R^59^ using ‘pROC’^60^ and ‘ROCR’^61^ packages. Statistical significance was determined by Mann-Whitney U test.

The clinical characteristics of the HCC patients in this study were summarized in Table 1. We investigated the association between plasma miRNAs and the various clinical characteristics in HCC patients from all the three cohorts. A normalization step was performed prior to the statistical analysis as described in the Supplemental Methods. The statistical tests for each of the clinical phenotypes are described below.

For binary clinical parameters e.g. gender (male and female), the differences in miRNA expression between the two groups were determined using Student’s t-test. Ordinal logistic regression test was conducted to assess the association between histological grade and each miRNA expression. Lastly, correlation between tumor size or serum AFP levels and miRNA expression was analyzed using Spearman’s rank correlation test. In all the statistical tests performed, Benjamini-Hochberg method was applied for multiple test correction, and corrected p-value < 0.05 was set as the significance threshold. All the statistical analyses above were performed in R.

The liver damage markers including AST, alkaline phosphatase (ALP), aminotransferase (ALT) and gamma-glutamyl transpeptidase (GGT), and the HCC marker, AFP were measured in the plasma samples of CHB, cirrhotic and HCC patients. Spearman’s rank correlation test was performed between the liver damage markers and each of the circulating miRNAs, followed by Benjamini-Hochberg correction. Circulating miRNAs with corrected p-value < 0.05 are considered as significantly associated.

Overall survival time was associated with plasma miRNA expression using the Cox proportional hazards test. Association between overall survival and clinical characteristics (Table 1) was also evaluated. Kaplan-Meier curves for the low- and high-expression groups (classified by the first and the third quartiles of the miRNA expression) were plotted. Circulating miRNAs with P-value < 0.05 by Cox proportional hazards test were considered to be significantly associated with overall survival. The ‘survival’ package^62,63^ in R was used for all the survival analyses.

### Ethical statement

The experimental protocols in this study were approved by SingHealth Centralized Institutional Review Board (CIRB Refs: 2013/455/B and 2014/823/B). All methods were carried out in concordance with relevant guidelines and regulations. Informed consent was obtained from all subjects and no participant was under 18 years old.

## Supplementary information

Supplemental Information

## Data Availability

The datasets generated during and/or analysed during the current study are available from the corresponding author on reasonable request.
