# Supplementary material for: Circulating microRNAs as Potential Diagnostic and Prognostic Biomarkers in Hepatocellular Carcinoma
Source: Sci Rep. 2019 Jul 18;9:10464. doi: 10.1038/s41598-019-46872-8 (PMC6639394; doi:10.1038/s41598-019-46872-8)
Supplement: Supplementary file 1 — Supplemental Information [file 41598_2019_46872_MOESM1_ESM.pdf]

Supplemental Information

Circulating microRNAs as Potential Diagnostic and Prognostic Biomarkers in Hepatocellular Carcinoma

Yu Jin[1], Ye Shen Wong [1], Brian K.P. Goh[2], Chung Yip Chan[2], Peng Chung Cheow[2], Pierce K.H. Chow[3,10], Tony K.H. Lim[4], George B.B. Goh[5], Thinesh Lee Krishnamoorthy[5], Rajneesh Kumar[5,10], Tze Pin Ng[6], Samuel S. Chong[7,8], Hwee Huang Tan[9], Alexander Y.F. Chung[2], London Lucien P.J. Ooi[2,10], Jason P.E. Chang[5], Chee Kiat Tan[5,*] and Caroline G. L. Lee [1,10,11,*]

***** Corresponding author

E-Mail: tan.chee.kiat@singhealth.com.sg, bchleec@nus.edu.sg

This file includes:

Supplementary Methods Page 1-4
Table S1. Page 5
Table S2 Page 6

Supplementary Methods

Inclusion criteria for NH, CHB and cirrhotic subjects

In collaboration with Health Sciences Authority, we recruited normal healthy donors as controls. The inclusion criteria were:

1. The subjects needed to be in good health and fulfilled the blood donor criteria by Health Sciences Authority. They were between 16 and 60 years old, with weight at least 45kg, haemoglobin level of at least 12.5g/dl, and did not have any symptoms of infection for at least 1 week.
2. Only Chinese subjects more than 30 years old were included, because the majority of the HCC patients were Chinese in the local cohorts (Table 2.2), and the age ranged from 30 to 83 years old.
3. We only included the subjects who were not infected by human immunodeficiency virus (HIV) or HBV, as determined by serological tests before blood donation.
4. Only subjects who declared no liver disease, HBV infection or cancer were recruited.

For CHB group, we collected blood samples from subjects who routinely visited Singapore General Hospital every three months and were tested positive for hepatitis B surface antigen. The hepatitis B carriers were symptomless carriers and did not develop HCC, NAFLD or cirrhosis within last six months. We recruited cirrhotic patients at SGH who were diagnosed of cirrhosis without CHB infection.

The sample collection from non-HCC individuals was accompanied by informed consent and approval from IRB (SingHealth IRB No: CIRB 2013/455/B) obtained before recruitment. Whole blood samples were obtained from 116 patients diagnosed of HCC in National Cancer Centre with approval from the Institutional Review Board (IRB) (NCC IRB No: NC08-12). Informed consents from the patients were obtained prior to blood collection.

Sample collection, processing, RNA isolation and quality control

Whole blood samples were collected in BD vacutainer® blood collection tubes containing EDTA and stored at 4°C in ice box immediately after collection. They were processed within one hour using the following steps. First, blood samples were centrifuged at 1900 ×g and 4°C for 10 minutes. The upper phase containing plasma was transferred to a clean falcon tube without disturbing the buffy coat phase. In order to remove cell debris, a high-speed centrifugation was then conducted at 16000 ×g and 4°C for 10 minutes. The clear supernatant was transferred to new microcentrifuge tubes and stored at -80°C.

For each sample, total RNA was extracted from 250 µl plasma using the miRCURY™ RNA isolation kit – biofluids (Exiqon, Vedbaek, Denmark). Plasma was thawed on ice and centrifuged at 3000 ×g and 4°C for 5 minutes in a microcentrifuge. An aliquot of 200 µl of plasma per sample was transferred to a new microcentrifuge tube and 60 µl of lysis solution BF containing 1µg carrier-RNA and RNA spike-in template mixture (including UniSp2, UniSp4 and UniSp5) was added to the sample. The tube was vortexed and incubated for 3 minute at room temperature, and 20 µl protein precipitation solution BF was added. The tube was then vortexed, incubated for 1 minute at room temperature and centrifuged at 11000 ×g for 3 minutes. The clear supernatant was transferred to a new collection tube, followed by addition of 270 µl isopropanol. The solutions were vortexed and transferred to a binding column. The column was incubated for 2 minutes at room temperature, and emptied using a vacuum-manifold. 100 µl wash solution 1 BF was added to the column. The liquid was removed using a vacuum-manifold, and 700 µl wash solution 2 BF was added. The liquid was removed using a vacuum-manifold again followed by addition of 250 µl wash solution and spinning of the column at 11000×g. The dry columns were transferred to a new collection tube and 50 µl RNase free H_2O was added directly on the membrane of the spin column. The column

was incubated for 1 minute at room temperature followed by centrifugation at 11000 ×g. The RNA was stored at -80°C.

Before profiling of genome-wide or candidate miRNAs in plasma samples, a quality control (QC) step was performed. Reverse transcription (RT) with 10 μl reaction volume was conducted on 2 ul total RNA using the miRCURY LNA™ Universal RT microRNA PCR, Polyadenylation and cDNA synthesis kit (Exiqon). Each RT was performed in duplicates, including an artificial RNA spike-in (UniSp6). We diluted the cDNA by 50× and profiled the miR-23a, miR-30c, miR-103, miR-142-3p, and miR-451 expression. Each miRNA was assayed once in a 10 μl reaction according to the miRCURY LNA™ Universal RT microRNA polymerase chain reaction (PCR) protocol. A negative control without template was included from the RT step, and profiled using all the QC assays. Reactions were performed in 384 well plates in a LightCycler® 480 Real-Time PCR System (Roche). The data were collected and analyzed using the Roche LC software, and the 2nd derivative method was used to determine the quantification cycle (Cq) values.

In the QC analysis, we identified consistent RNA extraction and RT efficiencies by evaluating the expression levels of the spike-in controls. Samples showing low spike-in signals were excluded and replaced with backup samples in the same biological groups. Hemolysis was assessed by the difference between Cq values of miR-23a and miR-451 in each sample, and the samples showing the difference more than 7 had a high risk of hemolysis and were replaced.

Data collection, processing and normalization

During the expression profiling using qPCR platform, the amplification efficiency was calculated using algorithms similar to the LinReg software and the raw Cq values were exported. Negative control evaluation was performed by removing the Cq values within 5 of

the negative control. In order to identify the best normalizer, NormFinder software [1] was used to evaluate the stability of the miRNAs expressed in all the samples. The global mean of all the commonly expressed miRNAs was also included. In both phases, the global mean of all the commonly expressed miRNAs outperformed any of the single miRNAs and was used as the normalizer. The normalized expression was then calculated using the following formula:

Normalized Cq = average Cq (Global mean) – assay Cq (sample)

In the first cohort, the global mean was calculated as the mean expression of the 109 commonly expressed miRNAs. Similarly, the global mean was based on the 59 common miRNAs in the second cohort and 68 common miRNAs in the third cohort.

For the association analysis with clinical characteristics, raw expression data from the three cohorts were pooled and an additional step of normalization was performed to eliminate the batch effect. The mean of all the 27 miRNAs that are expressed in all the 262 samples (29 in the first cohort, 106 in the second cohort and 127 in the third cohort) was calculated for each of the sample and used as the normalizer and the normalized expression was calculated using the same formula in the previous section by substituting the global mean as the average of the 27 common miRNA in all the samples of the two cohorts.

Table S1. Summary of samples recruited in the three cohorts.

Group	1st cohort (n=29)	2nd cohort (n=106)	3rd cohort (n=127)
Normal Healthy (NH)	10	40	29
Chronic HepB (CHB)		30	19
Cirrhotic			18
HCC	19	36	61

Table S2. Summary of association analysis for clinical characteristics and circulating miRNAs with overall survival by Cox proportional hazards test. Clinical characteristics or circulating miRNAs showing significant association with overall survival were labelled in bold.

Category	Predictor	hazard ratio	95% CI	p-value
**General**	age	0.99	0.95-1.02	4.19×10^{-1}
	**Viral Infection**	**2.76**	**1.08-7.08**	**3.44×10^{-2}**
	**AFP (>20ng/mL)**	**2.54**	**1.07-6.05**	**3.45×10^{-2}**
**Non-Tumor**	Normal liver cirrhosis	1.67	0.73-3.81	2.23×10^{-1}
	Steatosis	0.50	0.14-1.80	2.91×10^{-1}
	Dysplasia	1.68	0.47-6.08	4.27×10^{-1}
**Tumor**	**Histological Grade**	**2.27**	**1.19-4.35**	**1.31×10^{-2}**
	**Multifocality**	**2.88**	**1.29-6.44**	**9.74×10^{-3}**
	**TumorSize/cm**	**1.15**	**1.07-1.25**	**2.21×10^{-4}**
	Tumor Encapsulation	1.25	0.55-2.82	5.99×10^{-1}
	Incomplete Encapsulation	5.90	0.76-45.74	8.94×10^{-2}
	Tumor Present at Hepatic Capsule	1.43	0.49-4.17	5.13×10^{-1}
	Local Extension	2.50	0.74-8.40	1.38×10^{-1}
	Tumour Necrosis	2.45	0.91-6.56	7.56×10^{-2}
	Perineural Invasion	1.08	0.15-7.98	9.42×10^{-1}
	**Tumor Invasion**	**4.99**	**1.62-15.38**	**5.15×10^{-3}**
	**Vascular Invasion**	**3.51**	**1.58-7.79**	**2.09×10^{-3}**
**miRNA**	**miR-382-5p**	**1.64**	**1.26-2.14**	**2.87×10^{-4}**
	**miR-410**	**1.79**	**1.29-2.49**	**5.09×10^{-4}**
	**miR-139-5p**	**1.77**	**1.28-2.45**	**5.31×10^{-4}**
	**miR-101-3p**	**0.75**	**0.64-0.88**	**5.43×10^{-4}**
	**miR-424-5p**	**0.69**	**0.55-0.86**	**9.01×10^{-4}**
	**miR-128**	**2.35**	**1.4-3.94**	**1.14×10^{-3}**
